# Brief psychotic disorder treatment with Olanzapine in a patient with Phelan-McDermid syndrome

**DOI:** 10.1192/j.eurpsy.2024.1442

**Published:** 2024-08-27

**Authors:** I. Retsou, D. Antoniadis

**Affiliations:** ^1^4th Department of PICU, Psychiatric Hospital of Thessaloniki; ^2^Psychiatry Department, Aristotle University of Thessaloniki, Thessaloniki, Greece

## Abstract

**Introduction:**

The patient is a 50-year-old female, with multiple admissions in the PICU. At her first admission, at the age of 30 she presented the following main symptoms :mutism, negativism, crying and loss of bladder and bowel control. After collecting her complete family history, it was determined that her mother and one of her brothers were diagnosed with mild intellectual disability. Concerning her childhood history, she presented with late milestones as an infant and toddler and difficulties throughout primary education. Little information concerning her adult life was given, since the patient remained mute during the entirety of her first hospitalization.

**Objectives:**

Determination of the efficacy of olanzapine in a patient with Phelan-McDermit syndrome with mild intellectual disability and psychotic symptoms such as auditory hallucinations, delusional ideas and disrupted behavior.

**Methods:**

PANSS Test, intellectual capacity test, genetic testing.

**Results:**

PANSS Scale Score at the 1st day of admission:100

PANSS Scale Score at the last day: 79

Intellectual capacity test: mild intellectual disability

Genetic testing results: Phelan-McDermit syndrome

**Conclusions:**

After 20 days, symptoms showed mild recession in responce to 20mg of olanzapine. In a period of 12 months, the patient showed no signs of relapse and she was not readmitted in the PICU.

**Disclosure of Interest:**

None Declared

